# Supplementary material for: IFIT3 and IFIT5 Play Potential Roles in Innate Immune Response of Porcine Pulmonary Microvascular Endothelial Cells to Highly Pathogenic Porcine Reproductive and Respiratory Syndrome Virus
Source: Viruses. 2022 Aug 30;14(9):1919. doi: 10.3390/v14091919 (PMC9505468; doi:10.3390/v14091919)
Supplement: Supplementary file 1 [file viruses-14-01919-s001.zip › Table S1.pdf]

Table S1. Primer Pairs Used for Quantitative RT-PCR in this Study.

Target gene	Sequence (F: 5′-3′)	Sequence (R: 5′-3′)
IFIT3	CCGCCATCATGAGTGAGGTC	TCATGCCAGACATGTTCTTCCT
IFIT5	GAACCCAACCATCATGAGTGAA	AGAAATTCAAGCTGTTGCCCA
PRRSV N	ATCGCCCAACAAAACCAGTC	TGCGTCGGCAAACTAAACTC
IFN-a	GCCTCCTGCACCAGTTCTACA	TGCATGACACAGGCTTCCA
β-actin	GACCACCTTCAACTCGATCA	GTGTTGGCGTAGAGGTCCTT
