# Supplementary material for: IFIT3 and IFIT5 Play Potential Roles in Innate Immune Response of Porcine Pulmonary Microvascular Endothelial Cells to Highly Pathogenic Porcine Reproductive and Respiratory Syndrome Virus
Source: Viruses. 2022 Aug 30;14(9):1919. doi: 10.3390/v14091919 (PMC9505468; doi:10.3390/v14091919)
Supplement: Supplementary file 1 [file viruses-14-01919-s001.zip › Table S2.pdf]

Table S2. Specific siRNA Sequences of IFIT3 and IFIT5.

siRNA name	siRNA sequence (S: 5′-3′)	siRNA sequence (AS: 5′-3′)
si-IFIT3	GCAGUCCUCCGUGGAUAUUTT	AAUAUCCACGGAGGACUGCTT
	GGAGCUUGACUGUGAAGAATT	UUCUUCACAGUCAAGCUCCTT
	GCAAAUCAGCUUCCACAAATT	UUUGUGGAAGCUGAUUUGCTT
si-IFIT5	CCAAGAGACUUGCUCACAATT	UUGUGAGCAAGUCUCUUGGTT
	GGAAGAUACAAUUGGGCAATT	UUGCCCAAUUGUAUCUUCCTT
	GGUCUUCUCUGCGUACCAATT	UGGUACGCAGAGAAGACCTT
si-Negative Control	UUCUCCGAACGUGUCACGUTT	ACGUGACACGUUCGGAGAATT
